# Clinical presentation, prognostic factors, and outcomes of meningoencephalitis of unknown origin in older dogs

**DOI:** 10.1093/jvimsj/aalag089

**Published:** 2026-05-12

**Authors:** Frances E Anderson, Steven De Decker, R Timothy Bentley, Rita Gonçalves

**Affiliations:** Department of Veterinary Science, Small Animal Teaching Hospital, University of Liverpool, Leahurst Campus, Neston, Cheshire CH64 7TE, United Kingdom; Department of Veterinary Clinical Science and Services, Royal Veterinary College, University of London, Hawkshead Lane, North Mymms, Hatfield, Hertfordshire AL9 7TA, United Kingdom; Department of Veterinary Science, Small Animal Teaching Hospital, University of Liverpool, Leahurst Campus, Neston, Cheshire CH64 7TE, United Kingdom; Department of Veterinary Science, Small Animal Teaching Hospital, University of Liverpool, Leahurst Campus, Neston, Cheshire CH64 7TE, United Kingdom

**Keywords:** canine, encephalitis, immune-mediated, MUO, old

## Abstract

**Background:**

The clinical presentation and outcomes of older dogs with meningoencephalitis of unknown origin (MUO) have not been described previously.

**Hypothesis/Objectives:**

Compare clinical presentations and outcomes of an older cohort (≥8 years old) of dogs diagnosed with MUO compared to a younger cohort.

**Animals:**

Client-owned dogs diagnosed with MUO including 71 dogs ≥ 8 years old and 142 control dogs < 8 years old.

**Methods:**

Multicenter retrospective case–control study. Data were collected from the clinical records of 2 referral centers and referring veterinarians were contacted for additional survival data. Survival and relapse rates of the older and younger cohorts were compared.

**Results:**

On presentation, older dogs were found to be significantly more likely to have behavior changes (*P* = .01), cranial nerve deficits (*P* < .001), and comorbidities (*P* < .001) compared with younger dogs. On multivariate analysis, median survival time between the 2 groups did not differ significantly (16 months for older dogs and 24 months for younger dogs, *P* = .48). No significant difference was found in relapse rates between the 2 groups: 67/103 (65.0%) younger dogs and 20/48 (41.7%) older dogs relapsed within the study period.

**Conclusions and clinical importance:**

Older dogs with MUO were found to have similar survival times and relapse rates compared to younger dogs with MUO.

## Introduction

Meningoencephalitis of unknown origin (MUO) refers to a group of noninfectious, presumed immune-mediated, inflammatory diseases affecting the central nervous system (CNS) in dogs.^1,2^ Typically, a presumptive diagnosis is made based on clinical signs, advanced imaging, and cerebrospinal fluid (CSF) analysis, but a definitive diagnosis requires histopathology from biopsy or necropsy samples.^2^ Subtypes of MUO based on histopathology include granulomatous meningoencephalitis (GME), and necrotizing encephalitis, which can further be classified as necrotizing meningoencephalitis and necrotizing leukoencephalitis. Overlap potentially exists between these conditions.^3^

Studies have established a typical signalment for dogs with MUO as young-to-middle-aged, small and toy breed dogs, but dogs of any age and breed may be diagnosed with this condition.^4^ Granulomatous meningoencephalitis is most commonly diagnosed in dogs 4-8 years old, whereas necrotizing encephalitis typically has an even younger age of onset, with most dogs diagnosed before 4 years of age.^1,2,5^ Few studies have evaluated the association between age at diagnosis and outcome in dogs with MUO, and results have varied. One study including 52 dogs with MUO found that younger age was significantly associated with improved survival.^6^ A 2024 study investigating MUO in juvenile dogs (<52 weeks old) identified a median survival time (MST) of 84 days.^7^ A 2023 review evaluating 15 publications on MUO in dogs of any age reported a range of MSTs, primarily between 250 and 1834 days.^8^ Other studies have failed to identify any significant association between age at presentation and survival.^9,10^ Further investigations into the prognostic value of age in MUO and, more specifically, the clinical presentation and outcome of MUO in older dogs are currently unavailable.

The clinical characteristics and outcomes of an older cohort of dogs with MUO have not been evaluated previously. Our purpose, therefore, was to investigate the clinical presentation and outcomes of MUO in older dogs (≥8 years old at diagnosis) compared to a younger cohort of dogs. We aimed to compare clinical presentation, survival data, relapse rate, and response to standard immunomodulatory treatments between the 2 groups. By characterizing the disease in an older population, we sought to obtain crucial prognostic information to guide clinical decision-making and manage owner expectations for this challenging condition.

## Materials and methods

Cases and controls were identified from the University of Liverpool (UoL) and Royal Veterinary College (RVC) by searching their respective electronic databases for dogs diagnosed with MUO between 2008 and 2023. Dogs were categorized as late-onset MUO if they were 8 years of age or older at the time of diagnosis (older dogs), whereas the control group consisted of dogs that were less than 8 years old (younger dogs) at diagnosis. The cut-off of 8 years of age was decided because both small- and large-breed dogs of this age are considered senior or geriatric based on published literature.^11^ Twice as many controls as cases were identified using a random number generator to select control dogs.^12,13^ All dogs had an intracranial neuroanatomical localization (with or without concurrent spinal cord neurolocalization) on examination and a clinical diagnosis of MUO. Clinical findings supporting a diagnosis of MUO included magnetic resonance imaging (MRI) findings (intra-axial focal, multifocal, or diffuse lesions) with or without an increased cell count on CSF analysis (total nucleated cell count [TNCC] > 5/μL). Further inclusion criteria were: (1) a minimum follow-up time of 1 year (unless death occurred before that time); (2) complete medical records; and (3) negative infectious disease testing (when performed). The MRI examinations were performed using a 1.5 T (Philips Ingenia CX, Philips Healthcare) or a 1 T (Siemens Magnetom, Siemens Healthcare) scanner.

Data collected included patient signalment, duration, and progression of clinical signs, and examination findings including neurolocalization. Comorbidities present at the initial hospital visit for diagnosis also were recorded, and these were defined as the simultaneous presence of 2 health conditions in an individual.^14,15^ After a review of the clinical records, each case was assigned a neurodisability scale (NDS) score based on criteria previously described.^16^ Diagnostic results also were recorded, including MRI findings, CSF analysis results, and infectious disease testing. Treatment data included treatments administered at diagnosis as well as long-term treatments. Follow-up information was acquired by review of clinical records or contact with the referring veterinarian, and included time to relapse, death, or last contact. The cause of death was recorded as either MUO or another cause. Necropsy diagnosis was recorded when available.

The following data were used in the statistical analysis: age, sex, neuter status, breed, onset, duration, and progression of clinical signs before diagnosis, neurologic deficits and NDS score identified on presentation, neurolocalization (categorized as forebrain, brainstem, cerebellum, central vestibular [used when clinical signs were insufficient to localize as brainstem or cerebellum], optic nerves/chiasm or multifocal), CSF analysis results (TNCC and protein concentration), treatment, and survival to discharge. Onset of clinical signs was categorized as acute (<24 h), subacute (between 24 and 72 h), or chronic (>72 h). Initial treatment received was categorized as corticosteroids alone or corticosteroids and cytosine arabinoside (200 mg/m^2^ IV or SC), because no other initial treatments were administered. Long-term treatment was categorized as corticosteroids alone or corticosteroids and another immunomodulatory drug (eg, cytosine arabinoside, cyclosporine, or leflunomide). For statistical analysis, breeds with > 10 individuals represented were compared with the remainder of the study population.

Statistical analysis was performed using the software SPSS 29.0 (SPSS Inc., Chicago, Illinois). Continuous data were assessed for normality using the Shapiro–Wilk test. Descriptive statistics are reported for continuous variables using mean (SD) for approximately normally distributed variables and median (IQR) for variables with skewed distributions, and frequencies (with 95% CI where appropriate) are reported for categorical variables. Case and control dogs were compared using the χ-squared test for categorical variables and the Mann–Whitney test for continuous variables.

Univariable binary logistic regression was performed to identify variables associated with the different age groups. Before multivariable analysis, all variables were assessed for correlation using Spearman’s rank correlation coefficients. If Spearman’s rank correlation coefficient was > 0.8, the most statistically significant or biologically plausible variable was selected. Any independent variable demonstrating some association on preliminary univariable analysis (*P* < .2) was considered for inclusion in a multivariable model. Multivariable models were constructed using a manual backward stepwise removal approach; variables with *P* < .05 were retained. Cox proportional hazards analysis was used to identify clinical variables associated with long-term survival and relapse using the same approach. Survival times were assessed using Kaplan–Meier plots and were compared between groups by log-rank analysis.

## Results

Seventy-one cases of dogs with MUO 8 years or older at the time of diagnosis were identified after applying inclusion criteria (27 from UoL and 44 from RVC) and 142 control dogs were randomly selected from participating centers (56 from UoL and 86 from RVC). The demographic and clinical characteristics of the case and control dogs are summarized in Table 1.

**Table 1:** Demographic and clinical characteristics of the 213 dogs included in the study.

	Dogs < 8 years (*n* = 142)	Dogs ≥ 8 years (*n* = 71)	*P* value
**Median age (IQR)**	46 month (26-66)	112 months (103-122)	–
**Sex**			.77
** Female**	73 (51%)	35 (49%)	
** Male**	69 (49%)	36 (51%)	
**Neuter status**			<.001^a^
** Intact**	63 (44%)	13 (18%)	
** Neutered**	79 (56%)	58 (82%)	
**Median body weight (IQR)**	7.4 (4-10)	10.4 kg (6-20)	<.001^a^
**Onset of clinical signs**			.25
** Acute**	48 (34%)	26 (37%)	
** Subacute**	45 (32%)	15 (21%)	
** Chronic**	49 (34%)	30 (42%)	
**Median duration of clinical signs (IQR)**	7 days (3-14)	5 days (2-14)	.80
**Progression of clinical signs**	123 (87%)	53 (74%)	.03^a^
**Neurolocalization**			.49
** Forebrain**	49 (35%)	19 (27%)	
** Brainstem**	9 (6%)	6 (8%)	
** Cerebellum**	5 (4%)	3 (4%)	
** Central vestibular**	12 (8%)	8 (11%)	
** Optic nerves/chiasm**	2 (1%)	4 (6%)	
** Multifocal**	65 (46%)	31 (44%)	
**Clinical signs of raised ICP**	23 (16%)	5 (7%)	.07
**Median NDS score (IQR)**	6 (4-9)	4 (2-5)	<.001^a^
**Median CSF TNCC (IQR)**	39 cells/μL (12-280)	24 cells/μl (8-98)	.05^a^
**Median CSF protein concentration (IQR)**	0.46 g/L (0.25-0.92)	0.55 g/L (0.32-1.1)	.17

^a^Statistically significant.

Abbreviations: CSF = cerebrospinal fluid; ICP = intracranial pressure; NDS = neurodisability scale; TNCC = total nucleated cell count.

Forty-five breeds were represented. The most common breed identified was crossbreed (*n* = 31), followed by Chihuahua (*n* = 19), Maltese and West Highland White Terrier (both *n* = 17), and French Bulldog (*n* = 15). There were no Pug dogs in the older group, but there were 11 in the younger group. The neurologic abnormalities detected on initial presentation are presented in Table 2.

**Table 2:** Neurologic deficits identified on initial presentation in the 213 dogs included in the study.

	Dogs < 8 years (*n* = 142) (%)	Dogs ≥ 8 years (*n* = 71) (%)	*P* value
**Postural response deficits**	113 (80)	56 (79)	.91
**Ataxia**	93 (65)	49 (69)	.61
**Obtundation**	95 (67)	39 (55)	.09^a^
**Visual deficits**	70 (49)	25 (35)	.05
**Paresis**	58 (41)	21 (30)	.11^a^
**Cranial nerve deficits**	47 (33)	42 (59)	<.001^a^
**Seizures**	54 (38)	20 (28)	.15^a^
**Behavior changes**	37 (26)	25 (35)	.17^a^
**Head tilt**	40 (28)	19 (27)	.83
**Nystagmus**	33 (23)	13 (18)	.41
**Head turn**	34 (24)	6 (8)	.01^a^
**Tremors**	10 (7)	4 (6)	.70

^a^ *P* < .2 so included in the multivariable model.

All dogs except for one underwent an MRI; one younger dog had no advanced imaging because of a cardiopulmonary arrest that occurred before further investigations (this dog had a necropsy confirming a diagnosis of necrotizing meningoencephalitis). The majority of dogs had multiple lesions identified on MRI (158 dogs), whereas 46 had a single lesion. Three older dogs and 5 younger dogs had normal MRI scans, but these all had increased CSF TNCC. Cerebrospinal fluid was not collected because of concerns of increased intracranial pressure in 43 dogs. One hundred and ten dogs had serology or PCR tests for *Toxoplasma gondii* and *Neospora caninum* or both, neither of which indicated infection. Fifty-four dogs had co-morbidities recorded, including 29 older dogs and 25 younger dogs. The most common comorbidities were aspiration pneumonia (*n* = 11), ocular conditions (eg, corneal ulcers; *n* = 9), orthopedic conditions (eg, osteoarthritis; *n* = 8), and cardiac conditions (eg, mitral valve disease; *n* = 8). Necropsy examination was conducted for 14 dogs (8 younger and 6 older), a diagnosis of GME in 6 cases, necrotizing encephalitis in 5 cases, and 2 were considered to have MUO without a clear subtype described. One case had a necropsy examination, but the report was not available for review.

Fifty-two dogs received only corticosteroids at the time of diagnosis and 161 dogs received corticosteroids and cytosine arabinoside (95 IV and 66 SC). Long term, 31 dogs received corticosteroids only, whereas 130 received corticosteroids along with another immunosuppressive medication. Additional immunosuppressive medications included cytosine arabinoside (*n* = 122), cyclosporine (*n* = 23), leflunomide (*n* = 7), azathioprine (*n* = 6), procarbazine (*n* = 2), and lomustine (*n* = 1). No significant difference was found between initial treatment (*P* = .12) and long-term treatment (*P* = .85) among the different age groups.

On univariable logistic regression analysis, weight, breed, comorbidities, progression of clinical signs, clinical signs of increased intracranial pressure (ICP), epileptic seizures, obtundation, behavior changes, visual deficits, cranial nerve abnormalities, paresis, and NDS score showed some evidence of association with a diagnosis of late-onset MUO. However, on multivariable analysis, only the presence of behavior changes (*P* = .01; odds ratio [OR] = 2.86; 95% CI, 1.35-6.06), cranial nerve abnormalities (*P* < .001; OR = 3.14; 95% CI, 1.61-6.14), comorbidities (*P* < .001; OR = 3.78; 95% CI, 1.77-8.08), and lower NDS score (*P* < .001; OR = 0.72; 95% CI, 0.64-0.83) at presentation were associated with a diagnosis of late-onset MUO.

Median follow-up time was 13 months (IQR, 1-31). No significant difference was found between the follow-up times for younger or older dogs (*P* = .52). One hundred and sixty-seven (78%) dogs survived to discharge (79% of older dogs and 78% of younger dogs). Ninety-one dogs were alive at the time of last follow-up (37% of older dogs and 47% of younger dogs). Of the dogs that died during the study period, cause of death was unknown for 11 older dogs and 2 younger dogs. Nine older dogs and 5 younger dogs were euthanized for a condition unrelated to MUO. Using Kaplan–Meier analysis, the estimated median survival for dogs with MUO less than 8 years of age was 24 months (95% CI, 0-58) and 16 months (95% CI, 7-25) in older dogs; no significant difference in survival times was found among the age groups (*P* = .48). On univariable Cox regression, breed, center, progression of clinical signs, clinical signs of increased ICP, obtundation, epileptic seizures, paresis, comorbidities, CSF protein concentration, NDS score, and no administration of cytosine arabinoside at the time of diagnosis showed some evidence of association with death. On multivariable Cox regression analysis only obtundation (*P* = .002; OR = 2.07; 95% CI, 1.3-3.32), epileptic seizures (*P* = .002; OR = 1.85; 95% CI, 1.25-2.75), paresis (*P* < .001; OR = 2.25; 95% CI, 1.48-3.43), comorbidities (*P* = .01; OR = 1.72; 95% CI, 1.12-2.64), and lack of administration of cytosine arabinoside at time of diagnosis (*P* = .02; OR = 0.57; 95% CI, 0.37-0.9) remained associated with death.

Sixty-one dogs were excluded from relapse analysis because they either did not survive to discharge (46 dogs) or were euthanized within the first month because of insufficient improvement or progression of clinical signs (16 dogs). Relapse occurred in 87 of the remaining 152 dogs (57.2%; median time to relapse, 8 months; IQR, 2-15). These included 67/103 (65.0%) younger dogs and 20/48 (41.7%) older dogs. On univariable Cox regression, age group, weight, breed category, progression of clinical signs, obtundation, head tilt, nystagmus, paresis, CSF protein concentration, and NDS score showed some evidence of association with relapse. However, on multivariable Cox regression analysis only a higher NDS score (*P* = .01; OR = 1.11; 95% CI, 1.02-1.2) and breed (*P* = .02) remained associated with relapse. Breeds at higher risk of relapse were Yorkshire Terrier (*P* = .01; OR = 3.16; 95% CI, 1.39-7.1) and Pug (*P* = .003; OR = 5.07; 95% CI, 1.72-14.9). The late-onset group included no Pug dogs, but did include 4 Yorkshire Terrier dogs (3 of which relapsed).

## Discussion

We investigated the clinical presentation, prognostic factors, and outcomes of MUO in older dogs (≥8 years old) compared to a younger cohort. Older dogs diagnosed with MUO were found to be more likely to have behavior changes, cranial nerve deficits, and comorbidities compared with the younger dogs. Despite these differences, the age of presentation was not found to be significantly associated with either survival time (MST, 16 vs 24 months; *P* = .48) or risk of relapse.

The older cohort of dogs had a few differences in presentation compared to the younger dogs, including more dogs with behavior changes or cranial nerve deficits. The underlying causes for these changes are uncertain, particularly for cranial nerve deficits. A contribution of canine cognitive dysfunction (CCD) in the older dogs is a possible explanation for the increased frequency of behavior changes, considering that 14%-35% of dogs in this age group may be affected by CCD.^17^ The behavioral changes noted in CCD (eg, aimless wandering, inattentiveness, compulsivity, and excessive vocalization) are nonspecific and may overlap with clinical signs of MUO in some cases. In multiple sclerosis (MS) in human patients, cognitive impairment is frequent, with 40%-75% of patients experiencing some level, and it is more prevalent in older patients.^18^ This finding parallels our findings of increased behavioral changes in older dogs with MUO.

Similarities between MUO and MS in humans have been emphasized in the literature, because both are presumed to be immune-mediated idiopathic inflammatory CNS conditions. They share similar pathologic features including disruption of the blood–brain barrier, perivascular cuffing, and a tendency to affect CNS white matter, but MS more commonly involves demyelination, which is infrequently a component of MUO in dogs.^8,19–22^ In human medicine, more attention in recent years has been given to late-onset MS (LOMS), which is typically described as cases diagnosed after 50 years of age, and represents between 4% and 10% of MS diagnoses.^23,24^ These patients often present with a progressive disease course that is often faster than in younger patients, with relapses and disability often occurring sooner.^23,24^ Given the clinicopathological parallels between MS and MUO, further investigations into similarities between MUO in older dogs and LOMS in human patients may be warranted.

The older cohort of MUO patients was more likely to have comorbidities, emphasizing the challenge of managing concurrent health issues alongside a primary neurologic disease. Comorbidities have been found to be an important factor in human patients with LOMS, because patients diagnosed after age 50 are more likely to have multiple comorbidities including hypertension, hyperlipidemia, diabetes mellitus, intervertebral disc herniation, and ocular abnormalities.^23^ Older MS patients are also more likely to report trouble walking and urinary or fecal difficulties, and ultimately have a decreased survival prognosis.^23,25^ In dogs with MUO, concurrent conditions including aspiration pneumonia, urinary tract infections, renal failure, and gastrointestinal bleeding have been associated with increased mortality.^26–28^ At least some of these documented conditions are likely the result of MUO lesions or adverse effects of treatment and were seen in both the older and younger cohorts in our study. Overall, however, a larger proportion of older dogs (41.4%) had documented comorbidities compared with younger dogs (19.1%), reflecting the increasing prevalence of concurrent health conditions as dogs age. Ultimately, in both humans with LOMS and older dogs with MUO, co-existing health issues may worsen prognosis and increase the complexity of care required to manage the condition.

In our study, age was not found to be associated with either survival or relapse. In previous studies assessing the prognostic value of age in dogs with MUO, most have found similar results, identifying no association between age and poor outcome,^9,26,29–33^ except for a study that reported that younger age at diagnosis was associated with survival.^6^ This finding differs from that in humans with LOMS, who are described as having a worse prognosis, likely as a consequence of multiple factors related to the disease itself but also to advanced age (eg, comorbidities, higher incidence of complications, and difficulties in treatment).^24,34^ Despite age being associated with comorbidities in our study cohort, presence of comorbidities itself was not associated with outcome. This finding is in contrast to humans with LOMS, where comorbidities show a strong association with increased disability and death.^35,36^ The number and severity of comorbidities seen in aging humans and dogs show many similarities, but differ significantly in some areas (cardiac and vascular conditions are much more common in humans) and this finding may partially explain the difference in outcome between the 2 species.^37^

In the combined population of older and younger dogs in our study, multivariable analysis identified several significant risk factors for death, including obtundation, epileptic seizures, paresis, and the presence of comorbidities. These findings align with previous research in which decreased mentation and seizures were consistently associated with worse short-term survival in dogs with MUO.^9,31–33,38^ One study found that dogs that were obtunded at presentation had a 6.6-fold increase in the risk of death within the first week after diagnosis.^9^ In MUO, dogs with seizures previously have been documented to have a 2.3 times higher hazard ratio for death compared with dogs without seizures.^39^ Similarly, dogs without seizures have been found to have 4.2-fold increased odds of 1-week survival.^32^ These findings are not surprising, because seizures and decreased mentation likely indicate more severe or advanced disease, leading to a higher risk of early death or euthanasia.

Our results indicated that administration of cytosine arabinoside at diagnosis was associated with improved survival. This finding supports previous studies suggesting that prompt immunomodulation (eg, cytosine arabinoside) can improve early outcomes in severely affected dogs.^8,40–42^ However, research is conflicting because other studies have found no difference in outcome between dogs treated with cytosine arabinoside and corticosteroids compared to corticosteroids alone.^43,44^ Relatively few dogs received immunomodulatory drugs other than cytosine arabinoside and corticosteroids in our study, and it was not possible to investigate the effects of these other treatments. Prospective randomized clinical control trials would be necessary to fully evaluate the effects of MUO treatment on outcome.

Regarding relapse, a higher NDS score at presentation was significantly associated with an increased risk of future relapse, regardless of patient age. This finding highlights the NDS as a valuable tool for objectively quantifying clinical severity and predicting long-term disease course.^16^ Given this finding, it would be beneficial for clinicians to routinely utilize the NDS at diagnosis and during reassessment examinations, because it aids in determining prognosis and allows objective assessment of a dog’s neurologic status. In addition, it may provide useful information for future retrospective studies. Interestingly, NDS scores in the older cohort in our study were lower compared with the younger group. This finding may be a consequence of the presence of more dogs with breeds that are predisposed to necrotizing forms of MUO in the younger group, because these forms tend to have a rapidly progressive course and therefore severe disability at diagnosis.^5,45^ In addition, breed also proved to be a significant factor when investigating risk of relapse, with Yorkshire Terrier dogs and Pug dogs at a higher risk. This finding indicates that breed-specific genetic factors may influence disease progression. No Pug dogs were present in the older cohort. The median age of Pug dogs with necrotizing meningoencephalitis previously was found to be 18 months, and thus it is not surprising that older Pug dogs with MUO were not identified in our study.^5^

In human medicine, the Expanded Disability Status Scale (EDSS) is commonly used to measure degree of disability and track progression in MS patients.^46,47^ It has been found that older individuals with and without MS have higher EDSS scores, likely reflecting aging factors (eg, comorbidities) not directly related to MS.^46,48^ Although the NDS in dogs with MUO is meant to be used in a similar manner to the EDSS in MS patients, the clinical signs utilized by the NDS indicate obvious CNS dysfunction, and therefore aging dogs without clinically relevant neurological disease are unlikely to have a substantial NDS score.^16^ Conversely, the EDSS is much more detailed and records even subtle dysfunctions that can be communicated by human patients, including disabilities that commonly occur in an aging population without MS (eg, urinary urgency, mood alteration).^48^ Given the comparative simplicity of the NDS used in dogs, it is not surprising that a higher score indicating more severe disability correlates with a higher risk of relapse in dogs with MUO.

Our study had some limitations. Its retrospective, multicenter nature resulted in nonstandardized treatment protocols and potentially incomplete medical records. Some comorbidities may not have been documented or detected and therefore were not included in analyses. A definitive diagnosis of MUO requires histopathology, which was not available in most cases. Thus, the diagnosis for most cases remained presumptive and could not distinguish between GME and necrotizing subtypes. Furthermore, survival data can be influenced by the owner’s decision to euthanize, which may not solely reflect disease severity but also financial or quality-of-life considerations. It is also possible that many owners decline specialist referral for older dogs perceived to be nearing the end of life, particularly if the dogs have severe clinical signs, thus influencing the population of dogs studied. These limitations are common challenges in veterinary neurology research and emphasize the need for prospective studies. In addition, a future study with larger sample sizes would be more robust and allow enhanced confidence in the findings.

In conclusion, our study provides an analysis of MUO in an older canine population compared to younger dogs. Older dogs were found to be significantly predisposed to exhibiting behavioral changes and cranial nerve deficits, as well as comorbidities at the time of diagnosis. Our study also challenges the presumption that MUO carries a worse prognosis with age, because no significant difference was found in survival times or relapse rates between the 2 cohorts. Despite the differences observed in the presentation of older dogs, the only factors that influenced mortality or relapse were universal across both age groups, including obtundation, seizures, and paresis. Future research is warranted that focuses on prospective trials to establish optimal treatment protocols and further explores the specific pathophysiologic mechanisms that drive disease progression in aging dogs.

## Off-label antimicrobial declaration

The authors declare no off-label use of antimicrobials.

## Institutional animal care and use committee or other approval declaration

Approved by the Royal Veterinary College, University of London institutional ethical review board (URN: SR2024-01092006) and by the Veterinary Research Ethics Committee of the University of Liverpool (VREC1303).

## Abbreviations

CCD: canine cognitive dysfunction
CNS: central nervous system
CSF: cerebrospinal fluid
EDSS: Expanded Disability Status Scale
GME: granulomatous meningoencephalitis
ICP: intracranial pressure
LOMS: late-onset multiple sclerosis
MRI: magnetic resonance imaging
MS: multiple sclerosis
MST: median survival time
MUO: meningoencephalitis of unknown origin
NDS: neurodisability scale
OR: odds ratio
RVC: Royal Veterinary College
TNCC: total nucleated cell count
UoL: University of Liverpool
